# The alcohol dehydrogenase gene family in sugarcane and its involvement in cold stress regulation

**DOI:** 10.1186/s12864-020-06929-9

**Published:** 2020-07-29

**Authors:** Weihua Su, Yongjuan Ren, Dongjiao Wang, Yachun Su, Jingfang Feng, Chang Zhang, Hanchen Tang, Liping Xu, Khushi Muhammad, Youxiong Que

**Affiliations:** 1 Key Laboratory of Sugarcane Biology and Genetic Breeding, Ministry of Agriculture, Fujian Agriculture and Forestry University, Fuzhou, 350002 Fujian China; 2 Key Laboratory of Genetics, Breeding and Multiple Utilization of Crops, Ministry of Education, College of Crop Science, Fujian Agriculture and Forestry University, Fuzhou, 350002 Fujian China; 3 Department of Genetics, Hazara University, Mansehra, Pakistan

**Keywords:** Sugarcane, Alcohol dehydrogenases (*ADH*), Cold tolerance, ROS homeostasis, Evolution, Genome-wide analysis

## Abstract

**Background:**

Alcohol dehydrogenases (ADHs) in plants are encoded by a multigene family. ADHs participate in growth, development, and adaptation in many plant species, but the evolution and function of the *ADH* gene family in sugarcane is still unclear.

**Results:**

In the present study, 151 *ADH* genes from 17 species including 32 *ADH* genes in *Saccharum spontaneum* and 6 *ADH* genes in modern sugarcane cultivar R570 were identified. Phylogenetic analysis demonstrated two groups of *ADH* genes and suggested that these genes underwent duplication during angiosperm evolution. Whole-genome duplication (WGD)/segmental and dispersed duplications played critical roles in the expansion of *ADH* family in *S. spontaneum* and R570, respectively. *ScADH3* was cloned and preferentially expressed in response to cold stress. *ScADH3* conferred improved cold tolerance in *E. coli* cells. Ectopic expression showed that *ScADH3* can also enhance cold tolerance in transgenic tobacco. The accumulation of reactive oxygen species (ROS) in leaves of transgenic tobacco was significantly lower than in wild-type tobacco. The transcript levels of ROS-related genes in transgenic tobacco increased significantly. *ScADH3* seems to affect cold tolerance by regulating the ROS-related genes to maintain the ROS homeostasis.

**Conclusions:**

This study depicted the size and composition of the *ADH* gene family in 17 species, and investigated their evolution pattern. Comparative genomics analysis among the *ADH* gene families of *S. bicolor*, R570 and *S. spontaneum* revealed their close evolutionary relationship. Functional analysis suggested that *ScADH3*, which maintained the steady state of ROS by regulating ROS-related genes, was related to cold tolerance. These findings will facilitate research on evolutionary and functional aspects of the *ADH* genes in sugarcane, especially for the understanding of *ScADH3* under cold stress.

## Background

Plant metabolite production is affected by both plant development and environmental factors. Alcohols are components of plant volatiles and also act as plant stress signaling molecules [1]. Alcohol dehydrogenase (ADH, alcohol: NAD^+^ oxidoreductase, EC 1.1.1.1) acts as a dimer that relies on NAD(P) co-factors to interconvert ethanol and acetaldehyde and other short linear alcohols/aldehyde pairs [2]. *ADHs* are involved in seed development [3, 4], fruit development [5], and aerobic metabolism in pollen grain [6]. They also help to protect plants from flooding [7], drought [8], cold [9], and salt [10] stresses.

Plant alcohol dehydrogenase enzyme (ADH-P) was discovered to be active during hypoxia [11]. *ADH* gene activity is found at all stages of plant growth and under various stress conditions [10]. The *ADH* gene is involved in different aspects of plant growth and development [12, 13]. Several *ADH* genes are expressed in plant tissues in a developmentally-regulated manner, especially during fruit ripening. For example, *Le-ADH2* is involved in aroma volatilization during fruit ripening [12] . Overexpression of *Le-ADH2* improves fruit flavor by increasing alcohol levels (especially Z-3-hexenol) [12]. Tesniere and Verries et al. [13] found that *Vv-ADH1* and *Vv-ADH3* transcripts in grapes accumulate briefly in young fruits, while *Vv-ADH2* transcripts strongly increase in the mature phase named veraison. Expression of the *ADH* gene is induced by different environmental stresses [14], such as low temperature [9, 15], osmotic [16], drought [8], salt [10], mechanical damage [17], and the exogenous hormone abscisic acid (ABA) [18]. Dolferus et al. [18] found that hypoxia, dehydration, low temperature, and the phytohormone ABA can induce *ADH* expression in *Arabidopsis*. Shi et al. [14] demonstrated that alcohol dehydrogenase 1 gene (*ADH1*) enhances *Arabidopsis* resistance to abiotic and biotic stresses. Salinity stress induces accumulation of *ADH* mRNA in soybean (*Glycine max*) [19], grasspea (*Lathyrus sativus* L.) [20], and *Arabidopsis* [21]. The *ADH* gene is one of the most common cold-induced genes in cereal crops and *Arabidopsis* [9]. In addition, the *ADH* levels in octoploid strawberry are highly correlated with cold tolerance [15].

Sugarcane (*Saccharum* spp.) accounts for 80% of world sugar production [22]. Modern sugarcane cultivars are produced by hybridizations between *Saccharum officinarum* L. (2n = 80, high sugar content) and *Saccharum spontaneum* L. (2n = 40–128, disease resistance) [23]. However, adverse environmental factors such as drought, cold, salt, heavy metals, and low temperature can cause great production losses [24]. An effective solution is to identify genetic resources with superior genetic traits and then use molecular biology techniques to breed sugarcane cultivars resistant to stress [25]. However, the ploidy and repetitive genomic characteristics of sugarcane pose a challenge to sugarcane breeding. The development of genome-wide sequencing has aided resistance breeding. Two genomic datasets exist for *Saccharum*, one for the elite cultivar R570 [26] and the other for *S. spontaneum* [27].

Few studies have investigated the *ADH* genes in sugarcane. *ADH* gene expression upregulated due to waterlogging and waterlogging + nitrogen compounds in both leaf and root tissues [28]. *ADH* transcripts can also be induced by exogenous hormones (salicylic acid (SA), ABA, and methyl jasmonate (MeJA)) [29, 30]. However, the functions of sugarcane *ADH* genes are unknown. In the present study, we identified the *ADH* gene in 17 plant species including eudicots, monocots, basal angiosperms, and mosses. Phylogenetic analysis was used to study the evolutionary history and the origin of *ADH* genes in plants. We used bioinformatics to analyze the *ADH* genes in two sugarcane genomes for comparative analysis. These were the haploid genome of the modern sugarcane cultivar R570 [26] and the haploid genome version of the sugarcane ancestor *S. spontaneum* AP85–441 [27]. The *ADH* genes in the *Sorghum bicolor* genome, a common reference for comparative analysis of sugarcane [31], were also analyzed. We cloned and functionally characterized an *ADH* gene from sugarcane clones. Prokaryotic expression and ectopic expression studies showed that *ScADH3* is involved in the defense response to cold stress*.* These findings increase our understanding of the evolution and functional divergence of the *ADH* gene family in sugarcane and also the physiological mechanism and regulatory function of *ScADH3*. The data also provide insight into plant responses to cold.

## Results

### Identification of *ADH* genes in plant genomes

**Fig. 1:**
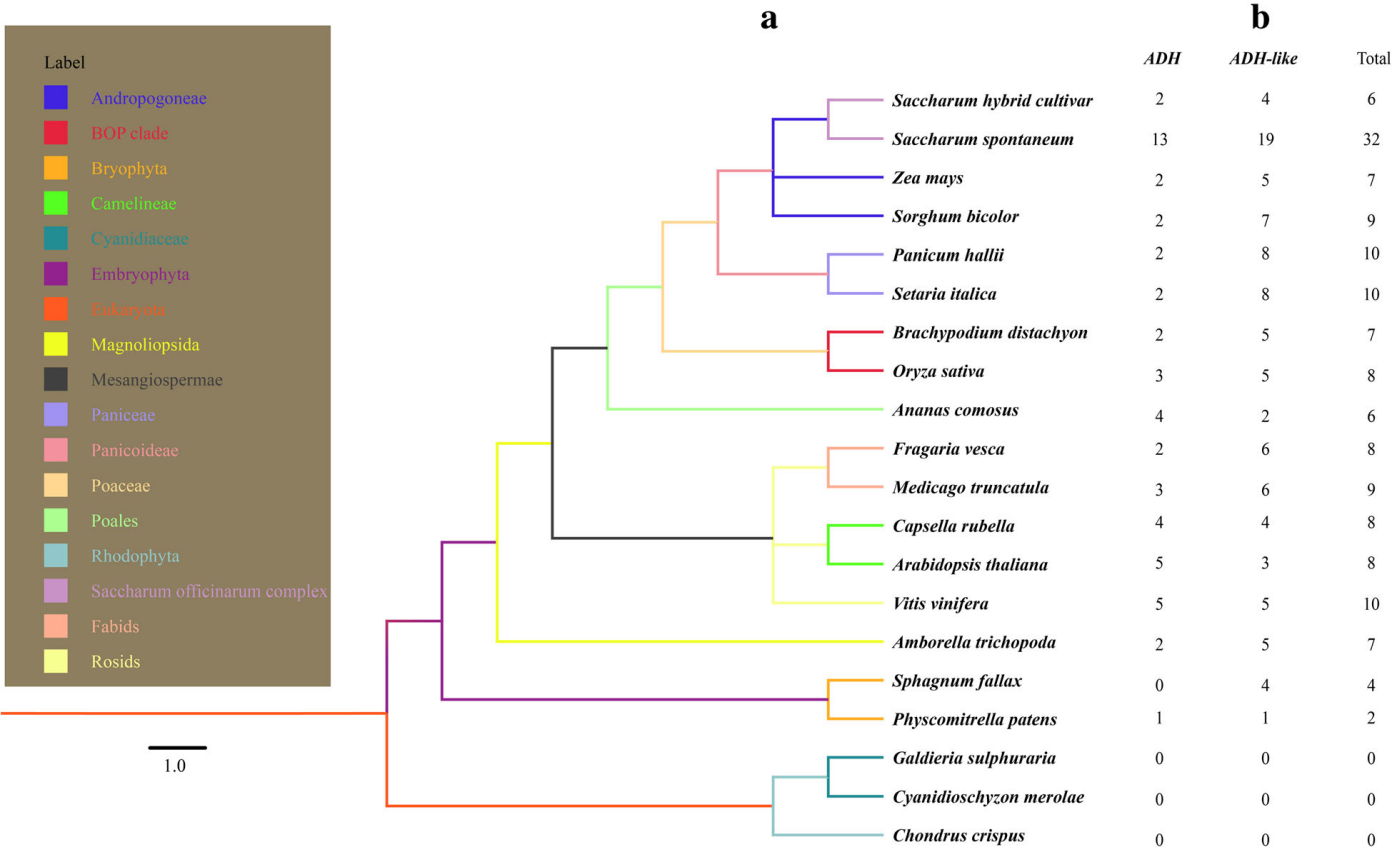
Genome-wide identification of ADHs in 20 species. **a** Common tree of 20 species used in this study. **b** The number of identified *ADH* genes in these 20 species

The putative ADH or ADH-like protein sequences were submitted to the CDD database, and 151 ADH protein sequences were retrieved from the 17 selected representative plant genomes. These included eudicots (5 species: 43 sequences), monocots (9 species: 95 sequences), basal angiosperms (1 species: 7 sequences), and mosses (2 species: 6 sequences). No ADH was identified in algae (Fig. 1 and Supplementary Table S1). The retained sequences that specifically hit the domain “alcohol_DH_plants (accession: cd08301)” were named ADHs, while the others were named ADH-likes. The copy number of *ADH* genes varied among the representative lineages of plants, ranging from two in *Physcomitrella patens* to 32 in *S. spontaneum*. The genes in *S. spontaneum* that belong to alleles [27] were designated as the same name followed by the letters “a,” “b,” “c,” and “d,” and duplicated genes were designated as the same name followed by the letters “e” and “f” [27]. Statistics results showed that eight copies of *ADH* genes are present in eudicots with the exception of *Medicago truncatula* which possesses nine *ADH* genes, and *Vitis vinifera*, which possesses 10 *ADH* genes. Among the monocots, the *ADH* gene copy number varied from 6 to 32. The largest number of *ADH* genes (32) was found in *S. spontaneum*. In the basal angiosperms, seven copies of *ADH* genes were identified in *Amborella trichopoda*. In the mosses, *P. patens* had two members of *ADH* genes, and there were four copies in *Sphagnum fallax*. However, no *ADH* genes were found in *Chondrus crispis*, *Cyanidioschyzon merolae* and *Galdieria sulphuraria*. One glutathione-dependent formaldehyde dehydrogenase (GSH-FDH) gene, also known as Class III ADH, was found in these three species. The *ADH-P* gene family is documented to have originated from Class III ADH [2]. Therefore, these Class III ADH genes were reserved for subsequent phylogenetic analysis.

Using the ExPASy ProtParam tool, the physical and chemical parameters of these ADH proteins were computed (Supplemental Table S1). The number of amino acids in ADH polypeptides ranged from 281 (SsADH-like4) to 881 (FvADH-like3), and the corresponding molecular weight (MW) ranged from 30.18 to 87.59 kDa. The computed theoretical isoelectric points (pI) varied greatly, ranging from 5.07 (AmADH-like3) to 8.82 (SfADH-like3). The results of the grand average of hydropathicity (GRAVY) suggested that 43 of 169 were hydrophilic and the instability index showed that 13 of 151 ADH proteins were unstable. Subcellular locations were predicted by WoLF PSORT program (Supplemental Table S1), and 102 ADH proteins were predicted to be located in the cytoplasm, while 30 ADH proteins were predicted to be localized in the chloroplasts.

### Phylogenetic classification of *ADH* gene family

**Fig. 2:**
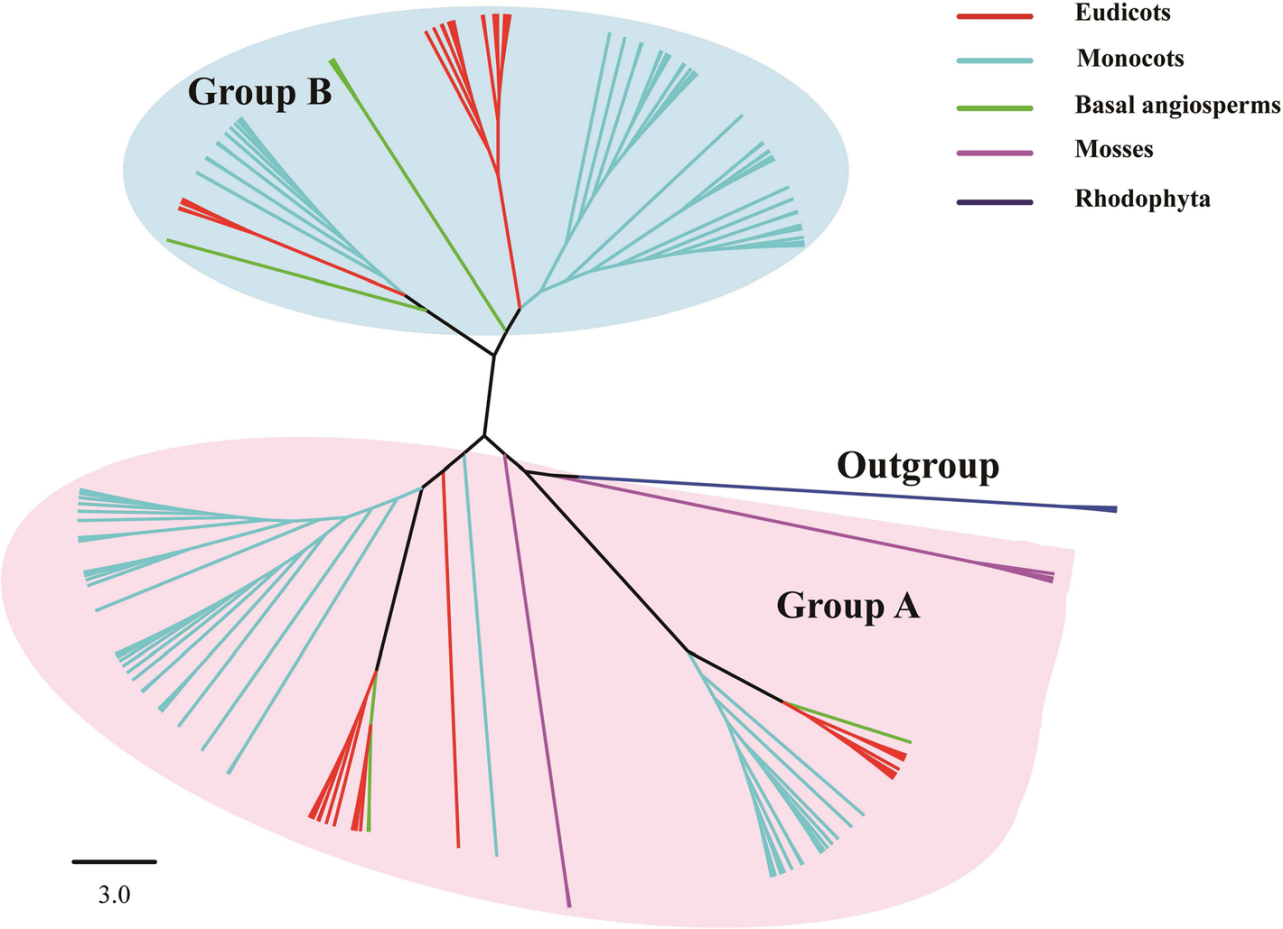
Phylogenetic classification of *ADH* genes in plants. Phylogenetic tree was constructed using the ML method implemented in RaxML-HPC2. The topology of the ML tree showed that ADH genes in plants can be classified into two major groups, which are designed as Groups A and B. The Rhodophyta was set as the outgroup

To study the evolution of the *ADH* gene family, a total of 153 ADHs from 17 plant species and three Class III ADHs from Rhodophyta as outgroups were used to construct a phylogenetic tree using the Maximum Likelihood (ML) method (Fig. 2 and Figure S1). Topology of the ML tree showed that *ADH* genes clearly cluster into two major groups in plants (Fig. 2), which were designated as Group A and Group B. In the plant clade, Group A possesses the whole land plant lineages, while the mosses were not located in Group B. The result implied that the *ADH* genes in Group A predated the *ADH* genes in Group B and had undergone several events of gene duplication during their long-term evolution. These gene duplication events generated multiple copies in plants.

The phylogenetic tree results demonstrated that the *ADH* gene family in plants can be classified into four subgroups, designated as Groups A-1, A-2, B-1, and B-2. Each subgroup can be further subdivided into mosses (Mos-1 and Mos-2), basal angiosperms (Bal-1, Bal-2, Bal-3, and Bal-4), monocots (Mon-1, Mon-2, Mon-3, Mon-4, and Mon-5), and eudicots (Eud-1, Eud-2, Eud-3, Eud-4, and Eud-5) (Figure S1). The Rhodophyta are separated from other embryophytes according to the topology of the ML tree. This may be because the *ADH* gene family has undergone one gene duplication event (D1) during the evolution of extant terrestrial and seed plants. The generation of four subgroups of the *ADH* gene family among plants may have been caused by the duplication event. Group A contains all embryophytes, while mosses are absent in Group B. Mosses form two branches in Group A, indicating that at least one gene duplication event occurred in the ancestor of mosses. The basal angiosperm (*A. trichopoda*) was distributed in each subgroup. The other seed plants also contain two monophyletic groups in Group A or Group B, respectively. These results suggest that gene expansion appeared in these plants during the evolution of the *ADH* gene family.

### Protein motifs and gene structure analysis

**Fig. 3:**
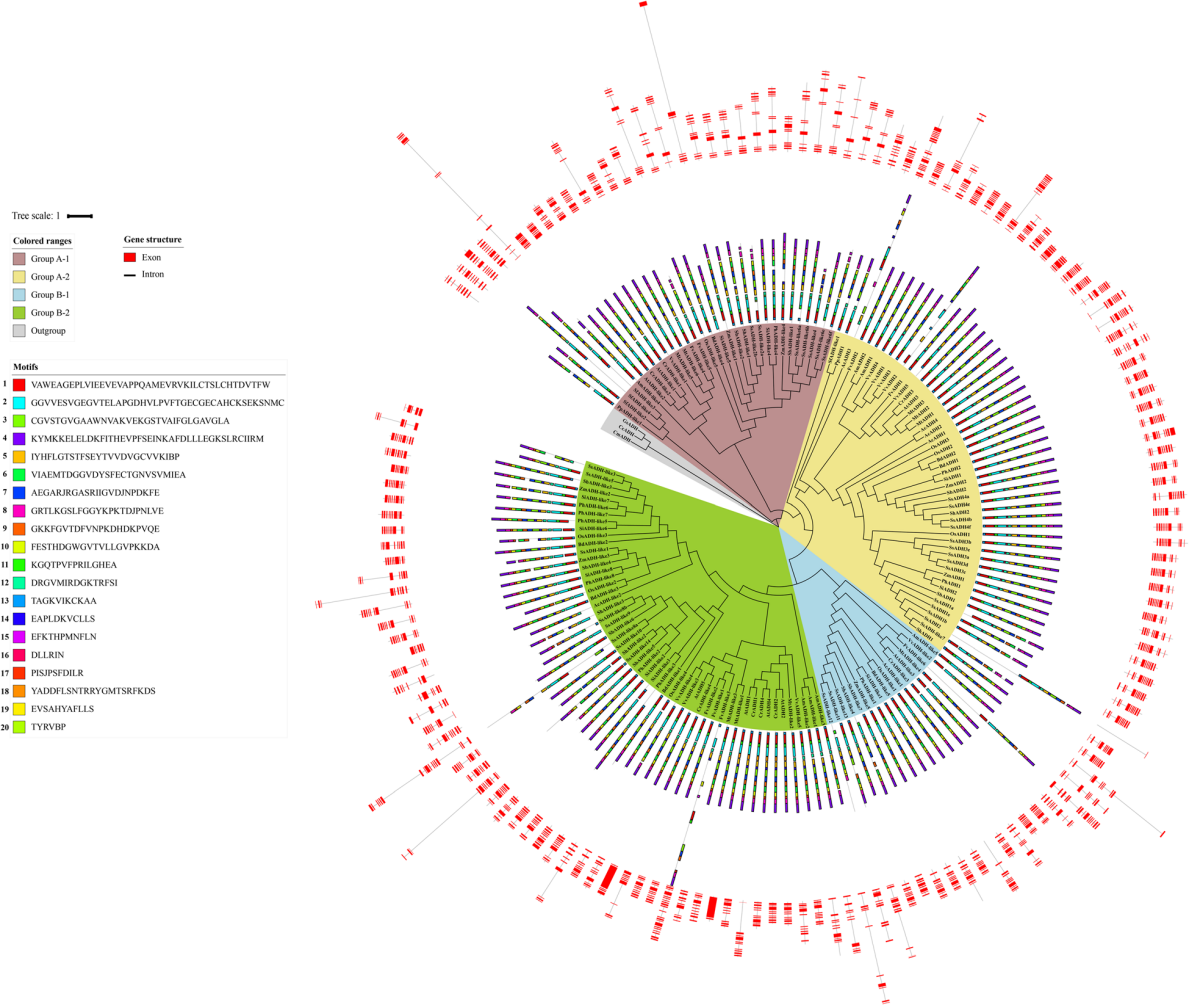
Phylogenetic relationships, gene structure and architecture of conserved protein motifs in *ADH* genes. The phylogenetic tree was constructed based on the full-length sequences of ADH proteins using RaxML-HPC tool. Details of clusters are shown in different colors. The motif compositions of ADH proteins. The motifs, numbers 1–20, are displayed in different colored boxes. Red boxes indicate exons in *ADH* genes, and black lines indicate introns

A schematic map representing the structure of all 151 ADH proteins from 17 species was constructed from the MEME motif analysis results (Fig. 3). A total of 20 distinct conserved motifs were found. Most ADH members were within the same clade, especially the most closely related members, which usually shared common motif compositions. This indicated potential functional similarities among the ADH proteins. However, some motifs were group-specific: motif 20 was unique to almost all Group A-1; motif 16 was unique to almost all A-2 members; motif 19 was unique to almost all Group B-1 members; motif 17 was unique to almost all Group B-2 members. Most copies of ADHs proteins possessed 15 or 16 motifs. There was no correlation between motif number and protein length. For example, SsADH-like4 had the shortest protein length but its motif number was not the smallest. FvADH-like3 had the longest protein length, and its motif number was not the largest.

The pattern of exon–intron distribution and position of the *ADH* genes from 17 species were analyzed to study the structural characteristics and evolution of the *ADH* gene family (Fig. 3). Most *ADHs* (90 of 151) contained 10 exons, and 30 of 151 *ADHs* had nine exons. The exon number of *ADHs* ranged from 1 to 21. In Group A-1, 16 of 31 *ADHs* contained 10 exons, 12 *ADHs* contained 8, 9, or 11 exons, and one *ADH* had 12 exons (Fig. 3). In Groups A-2 and B-1, the number of introns was conserved, 39 of 48 and 13 of 17 *ADHs* had 10 exons. These genes in Group B-2 contained 6 to 21 introns. These data showed that the intron number of *ADHs* in Group B-2 was variable.

### Chromosomal distribution and duplications to the family expansion

**Fig. 4:**
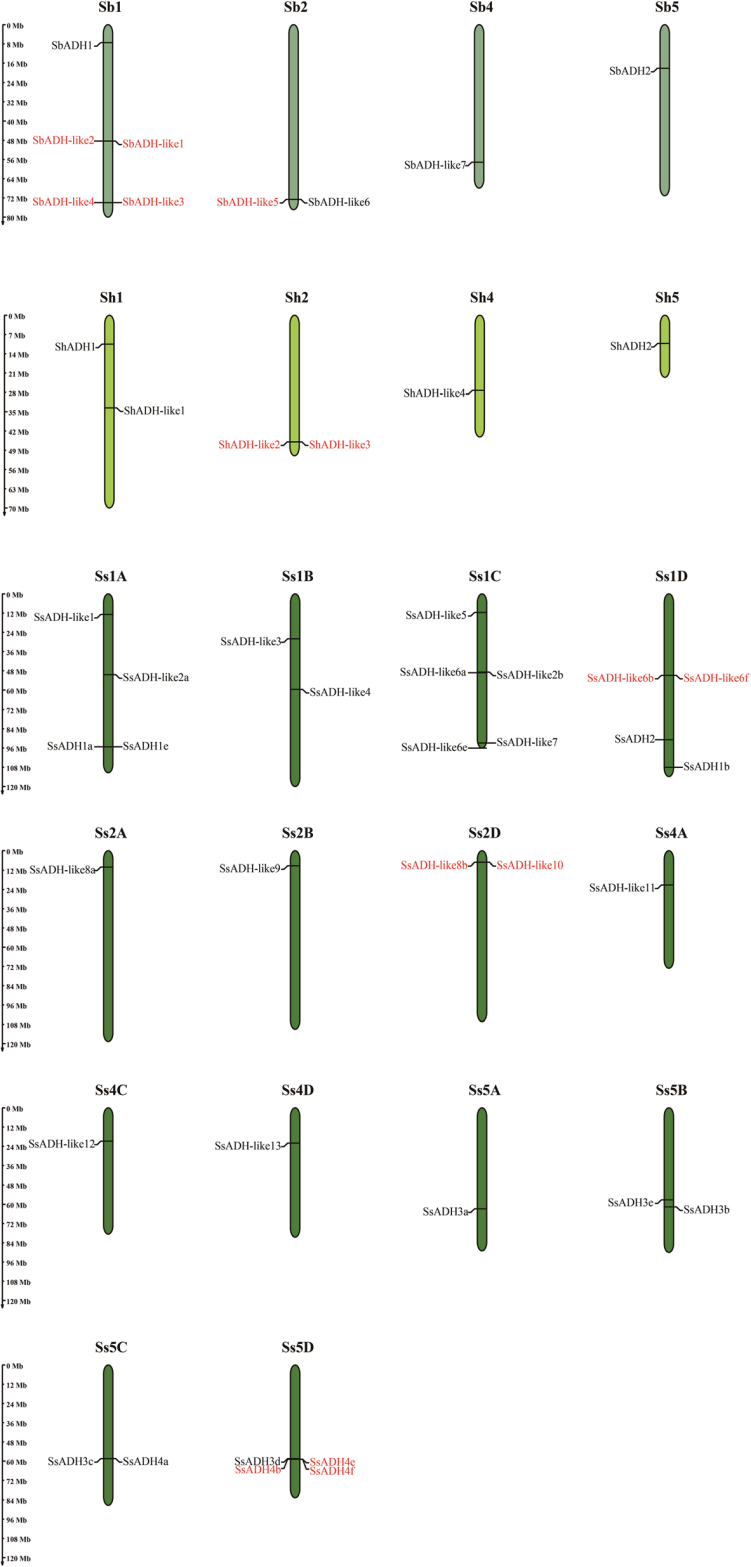
Chromosomal distribution of the *ADH* gene family in *S. bicolor*, *S. spontaneum* and R570. The tandem duplicated genes are represented by red font. The scale bar on the left indicates the chromosome length (Mb)

*SbADH* genes were unevenly distributed on four sorghum chromosomes (Fig. 4). The majority of *SbADH* genes were located on chromosome 1 (Sb1). The six *ShADH* genes were randomly distributed on four R570 chromosomes. Chromosome 1 (Sh1) and Chromosome 2 (Sh2) each contained two genes. The distribution of *SsADH* genes on the 14 chromosomes was uneven. The number of *SsADH* genes per chromosome varied from one to five (chromosomes 1C (Ss1C)). There was no correlation between chromosome length and the number of *ADH* genes.

**Fig. 5:**
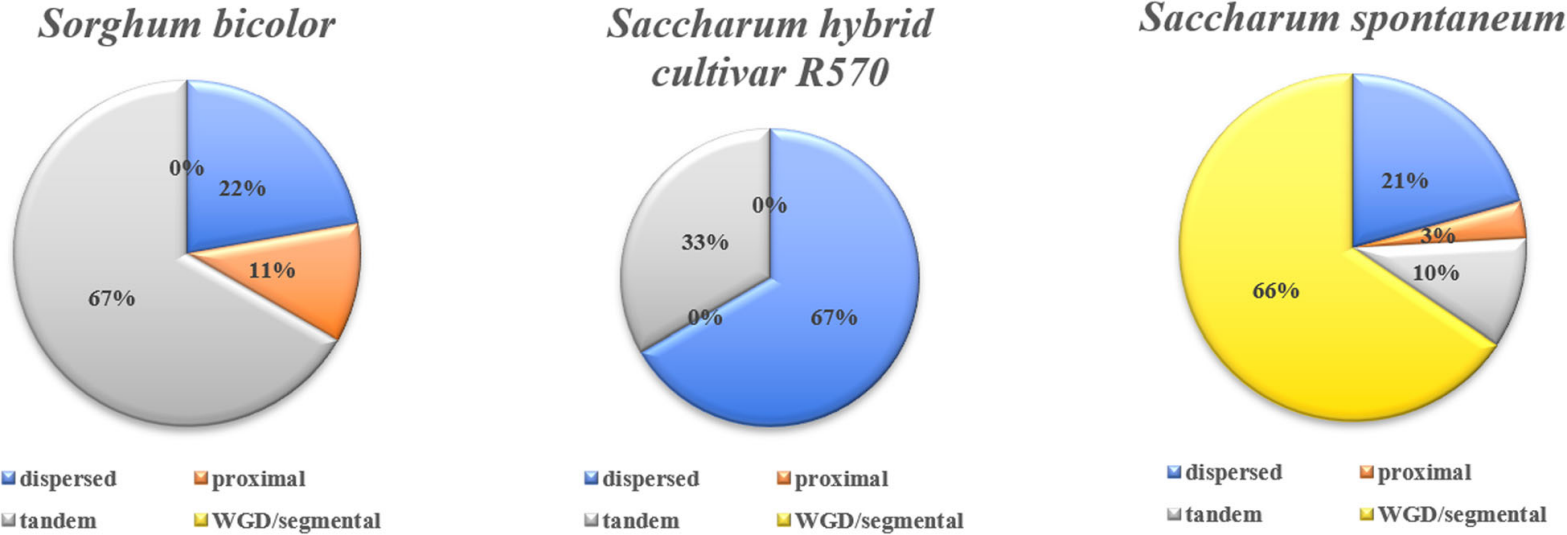
Expansion mechanisms of the *ADH* gene family in *S. bicolor*, R570, and *S. spontaneum*. The effects of dispersed, proximal, tandem, and WGD/segmental duplications on the expansion of ADH genes in *S. bicolor*, R570, and *S. spontaneum*

The types of *ADH* genes in *S. bicolor*, *S. spontaneum* and R570 were identified by the Multiple Collinearity Scan toolkit (MCScanX) software to study the possible gene expansion mechanisms (dispersed, proximal, tandem, and WGD/segmental duplications) in these species (Fig. 5 and Supplemental Table S2). Dispersed and tandem duplications were found in all three species. Proximal duplication was observed in *S. bicolor* and *S. spontaneum*, but only *S. spontaneum* contained the WGD/segmental duplication (Fig. 5). For *S. bicolor*, tandem duplication played an important role in the gene expansion because more than half of the *SbADHs* were related to tandem duplication. For R570, dispersed duplication played an important role in the gene expansion. However, for *S. spontaneum*, more than half of *SsADHs* were related to WGD/segmental duplication (Fig. 5). Although the rates of the four duplicated types in the three species varied, the dispersed and tandem duplications appear to be the most common mechanisms for *ADH* family gene expansion.

### Synteny analysis of ADH family genes

**Fig. 6:**
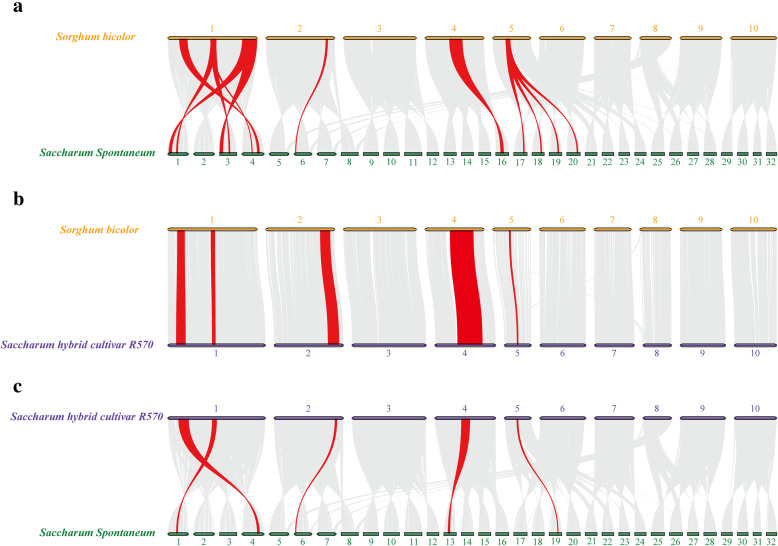
Synteny analysis of ADH genes in *S. bicolor*, R570, and *S. spontaneum*. **a** Synteny analysis of ADH genes in *S. bicolor* and *S. spontaneum*. **b** Synteny analysis of ADH genes in *S. bicolor* and R570. **c** Synteny analysis of ADH genes in R570 and *S. spontaneum*. Gray lines in the background indicate the collinear blocks within *S. bicolor*, R570, and *S. spontaneum*; red lines highlight the syntenic ADH gene pairs

Synteny analysis can help reveal the phylogenetic relations among ADH family genes. Three comparative syntenic maps between *S. bicolor*, R570, and *S. spontaneum* were constructed (Fig. 6 and Supplemental Table S3). A total of 12 *SsADH* genes showed a syntenic relationship with *S. bicolor* (Fig. 6a). Five *SbADH* genes showed a syntenic relationship with R570 (Fig. 6b). To some extent, the observed synteny between *ADH* genes of R570 and *S. bicolor* may be regarded as an outcome of the strategy adopted to assemble *Saccharum* BAC clones. There were 10 orthologous pairs between *S. spontaneum* and R570 (Fig. 6c). Two *SbADH* genes (*SbADH-like1* and *SbADH2*) were associated with three or four syntenic gene pairs within *S. spontaneum*. *ShADH* genes, *ShADH1*, *ShADH2*, *ShADH-like1*, and *ShADH4* were associated with two syntenic gene pairs within *S. spontaneum*. These results suggest that these genes may have been involved in the evolution of the *ADH* gene family. Comparing the syntenic blocks, 12 *ADH* collinear gene pairs (six pairs between *S. bicolor* and *S. spontaneum*, three pairs between R570 and *S. spontaneum*, and three pairs between *S. bicolor* and R570) were anchored to highly conserved syntenic blocks, which contain more than 100 genes. Only four *ADH* collinear gene pairs (two pairs between *S. bicolor* and *S. spontaneum*, one pair between R570 and *S. spontaneum*, and one pair between *S. bicolor* and R570) were located in syntenic blocks that possessed fewer than 30 orthologous gene pairs.

The Ka/Ks ratios of the *ADH* gene pairs were calculated to study the evolutionary constraints acting on the *ADH* gene family (Supplemental Table S3). Only the SsADH-like11/ShADH-like4 gene pair had Ka/Ks > 1. The other orthologous *ADH* gene pairs had Ka/Ks < 1, suggesting that the *ADH* gene family might have experienced strong purifying selective pressure during evolution.

### Identification and sequence characteristics of *ScADH3*

**Fig. 7:**
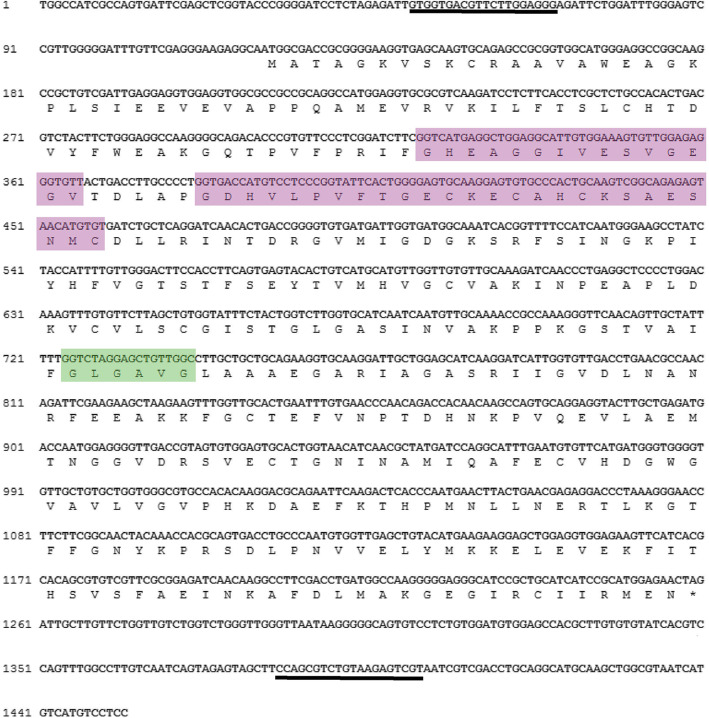
Complete cDNA and deduced amino acid sequences of the *ScADH3* gene. The full length cDNA was 1452 bp with an ORF encoding 379 amino acids. The purple-shaded portion shows the Zn binding motif, and the green-shaded portion indicates the NADPH binding motif. The underlined sequences show the specific amplification primer pair for *ScADH3*. * represents the stop codon

*AtADH1* (GenBank Acc No. NM_106362.3) is a commonly studied *ADH* gene. It plays a critical role in response to hypoxic conditions [32], low temperatures, [33] osmotic stress, [34], and pathogen infection [14]. In this study, the full coding sequence of *ScADH* (GenBank Acc No. MN_879279), which is homologous to *AtADH1*, was isolated from *Saccharum* spp. hybrid (ROC22) by reverse transcription-polymerase chain reaction (RT-PCR). Percentage of identity between 6 ShADHs, 32 SsADHs and 1 ScADH showed that this ScADH had high homology with SsADH3a (98.94%), SsADH3b (98.42%), SsADH3c (99.47%), SsADH3d (99.47%), and SsADH3d (98.14%) (Supplemental Table S4). This *ScADH* was designated as *ScADH3*. The length of the ORF in *ScADH3* was 1137 bp, and it encoded polypeptide of 379 amino acids (Fig. 7). The protein primary structure analysis showed that MW, pI, GRAVY, and instability index (II) of the ScADH3 protein were 40.82 kDa, 6.03, − 0.001, and 29.54. These values suggest that ScADH3 is an acid stable basic hydrophilic protein (Supplemental Table S5). The results of protein secondary structures showed that ScADH3 mainly consisted of a random coil (50.13%), alpha helix (25.07%), and extension chains (24.80%) (Supplemental Table S6). The amino acid sequence of ScADH3 contains a Zn1-binding signature [GHE(X)2G(X)5G(X)2 V], a Zn2 structural motif [GD(X)9,10C(X)2C(X)2C(X)7C], and an NADPH-binding domain [GXG(X)2G] motif (so-called Rossmannfold) (Fig. 7).

### Expression of *ScADH3* in response to biotic and abiotic stresses

**Fig. 8:**
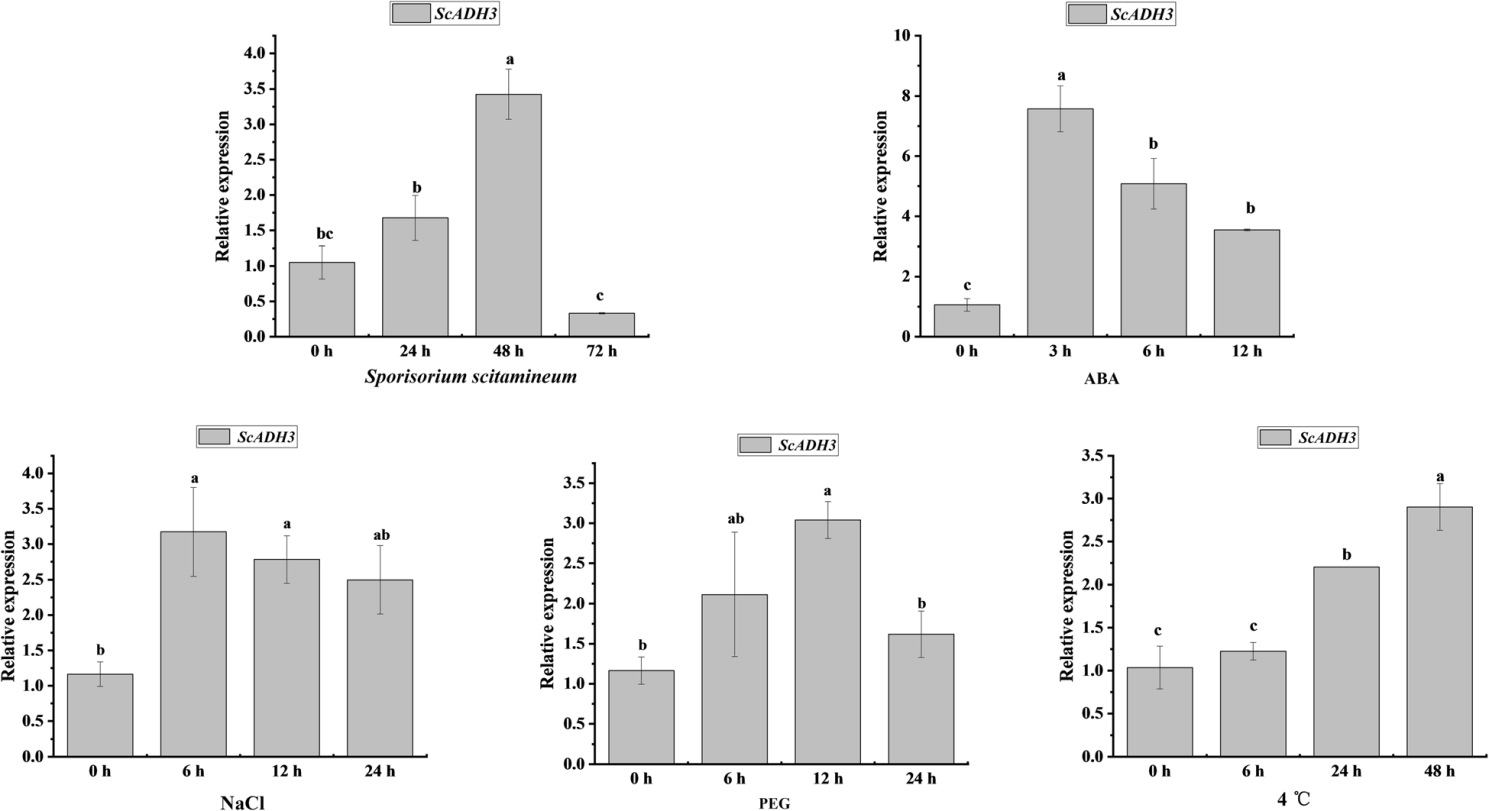
qRT-PCR expression analysis of the *ScADH3* gene in sugarcane ROC22 plantlets after treatment with *Sporisorium scitamineum*, 100 μM ABA, 25% polyethylene glycol (PEG) 8000, 250 mM NaCl, and 4 °C. The expression levels of *GAPDH* were used for normalization. All data points shown are mean ± SE (*n* = 3). Different lowercase letters indicate a significant difference, as determined by Duncan’s new multiple range test (*p* < 0.05)

Quantitative reverse transcription polymerase chain reaction (qRT-PCR) was used to detect the expression of *ScADH3* under *Sporisorium scitamineum*, ABA, sodium chloride (NaCl), polyethylene glycol (PEG), and cold (4 °C) stresses (Fig. 8). The expression of *ScADH3* was upregulated at 24 h and 48 h, but inhibited at 72 h under *S. scitamineum* stress. Under ABA and NaCl stresses, *ScADH3* was upregulated at all treatment time points. Under PEG stress, the transcript of *ScADH3* was induced at 6 h and 12 h. In response to 4 °C stress, the expression of *ScADH3* was upregulated at 24 h and 48 h. Of great interest is that the expression of *ScADH3* was upregulated at all time points in response to cold stress. Therefore, we were attempted to validate its function in prokaryotic (*E. coli*) and eukaryotic (*N. benthamiana*) cells in the following experiments.

### Overexpression of *ScADH3* enhances the cold tolerance of *E. coli* cells

**Fig. 9:**
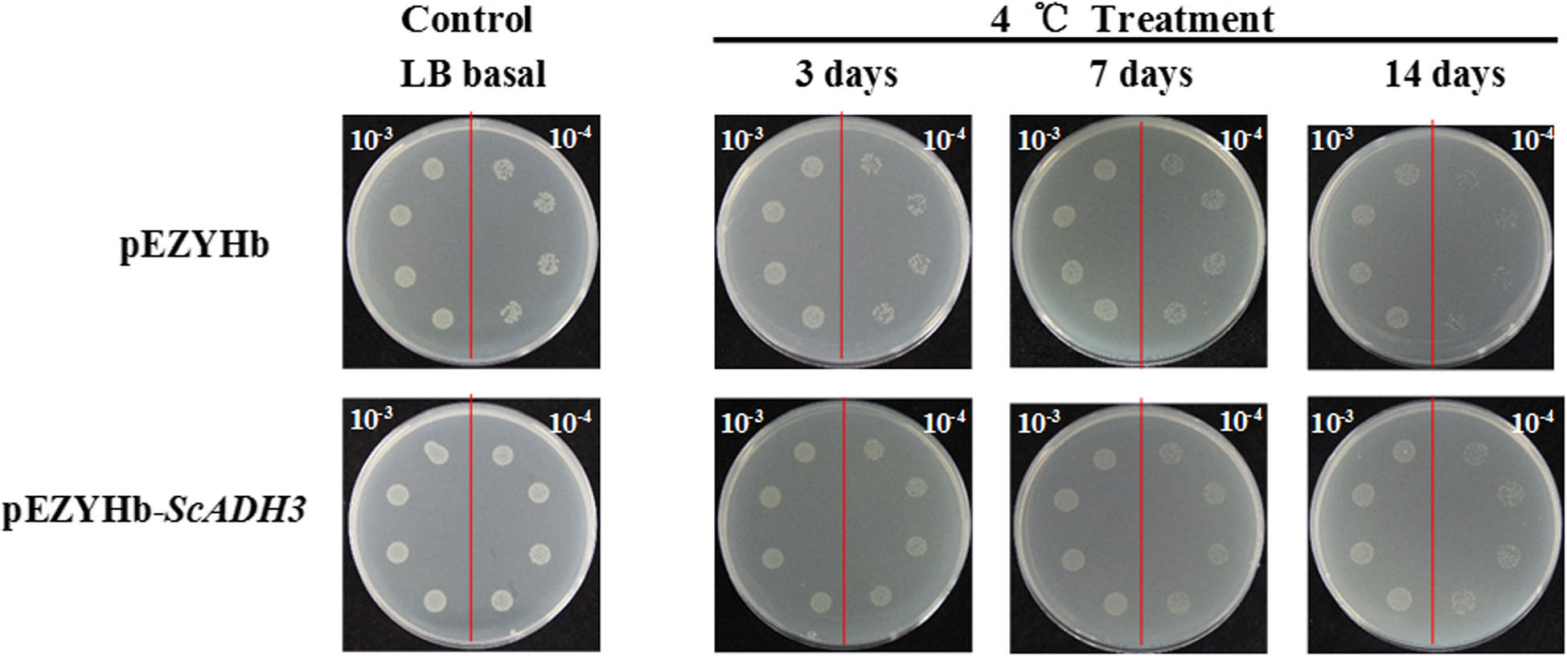
Spot assays used for monitoring the growth performance of BL21/pEZYHb and BL21/pEZYHb-*ScADH3* transformed *E. coli* cells on LB plates under cold stress. The growth performance of BL21/pEZYHb and BL21/ pEZYHb-*ScADH3* cells on LB plates without supplements or stresses were used as controls. After spotting the sample on LB agar plates, incubation at 4 °C in darkness for 3 days, 7 days, and 14 days was used to study the tolerance of BL21/pEZYHb and BL21/ pEZYHb- *ScADH3* cells under cold stress

*E. coli* cells were transformed with the empty vector of pEZYHb or the recombinant vector of pEZYHb-*ScADH3* and used to study the tolerance of *ScADH3* to cold stress (Fig. 9). In normal conditions, the *E. coli* cells of pEZYHb or pEZYHb-*ScADH3* showed similar and normal growth on solid LB medium. Under cold stress, pEZYHb-*ScADH3-*transformed bacterial cells with 10^− 4^-fold dilutions had increased numbers and better survival compared to the untransformed cells, especially after 14 days treatment. These results suggested that *ScADH3* may help enhance the tolerance of *E. coli* cells to cold stress.

### Overexpression of *ScADH3* improves the cold tolerance of transgenic *N. benthamiana*

**Fig. 10:**
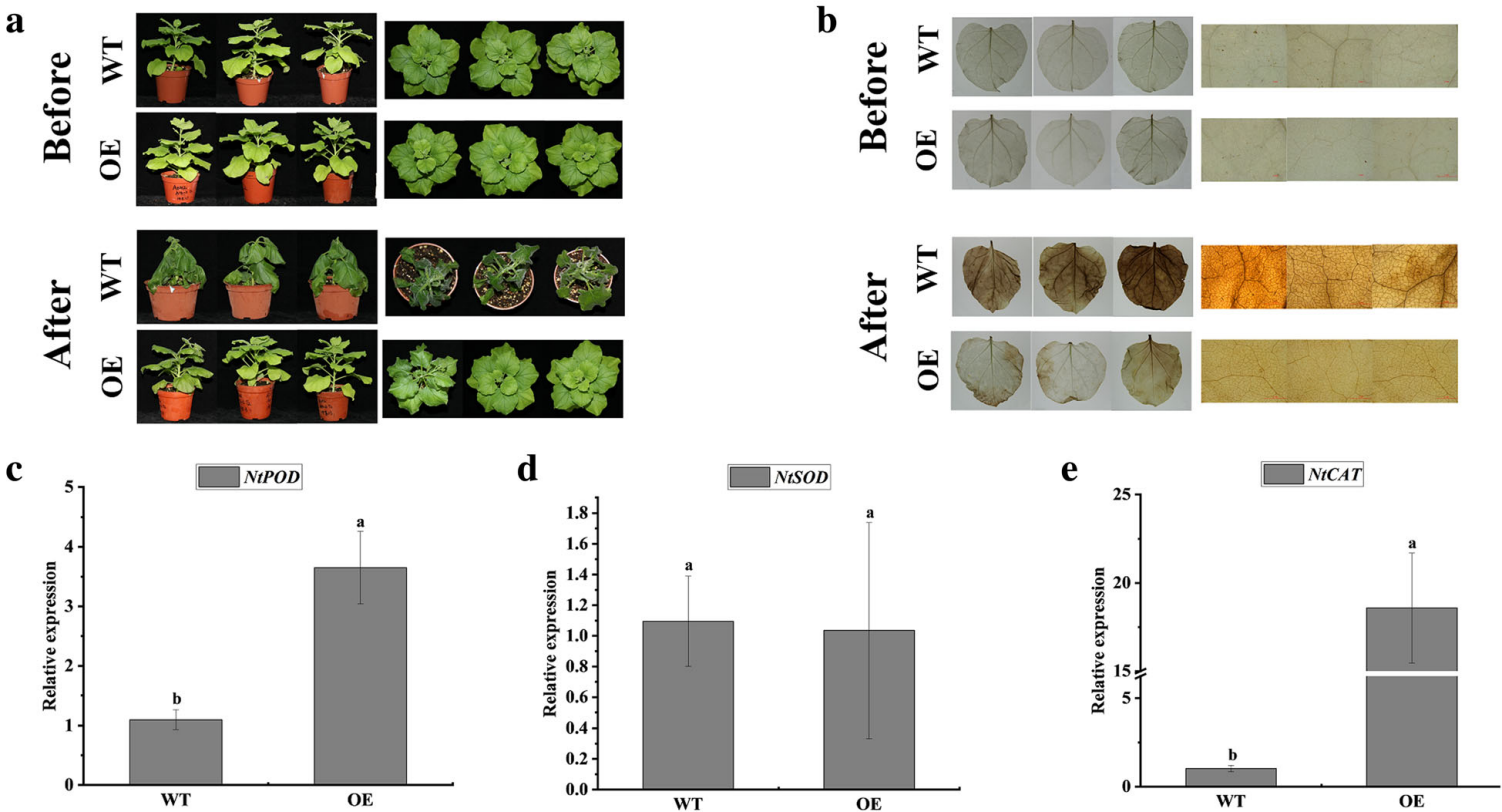
Overexpression of *ScADH3* enhances the cold tolerance of transgenic tobacco. **a** Phenotypes of 2-month-old plants of transgenic line (OE) and the wild type (WT) before and after freezing treatment (− 2 °C for 3 h). **b** Histochemical staining with DAB (upper panels) for detection of in situ accumulation of H_2_O_2_ in the transgenic lines (OE) and the wild type (WT) before and after cold treatment. **c**-**e** are qRT-PCR expression analysis of *NtPOD* (**c**), *NtSOD* (**d**), and *NtCAT* (**e**) in the (OE) and the wild type (WT). *Ubiquitin* was used as the reference gene. All data points shown are mean ± SE (*n* = 3). Different lowercase letters indicate a significant difference, as determined by Duncan’s new multiple range test (*p* < 0.05)

Transgenic *N. benthamiana* overexpressing *ScADH3* was used for additional analysis of *ScADH3* function in cold tolerance. The phenotypes of wild type (WT) and transgenic *N. benthamiana* were similar (Fig. 10a)*.* However, in the cold treatment, the WT had a more severe water-soaking phenotype than the transgenic plants. Excessive ROS will cause oxidative stress, which will negatively affect cell integrity [35]. Histochemical staining showed that the WT leaves showed deeper staining by DAB than those of the transgenic plants (Fig. 10b). This indicates that the transgenic plants had lower levels of ROS in response to cold stress. To study the molecular mechanisms underlying the enhanced cold tolerance by overexpressing *ScADH3*, qRT-PCR was used to detect the mRNA abundance of the ROS-related genes *NtSOD*, *NtPOD*, and *NtCAT* in the WT and transgenic lines. Except for *NtSOD*, transcript levels of *NtPOD* and *NtCAT* were higher in the transgenic lines than in the WT (Fig. 10c–e), indicating that overexpression of *ScADH3* led to an up-regulation of the ROS-related genes. These results demonstrated that overexpression of *ScADH3* improved cold tolerance of the transgenic tobacco plants.

## Discussion

Alcohol dehydrogenases (ADHs, alcohol: NAD^+^ oxidoreductase, EC 1.1.1.1) can utilize NADH as a reducing agent to catalyze the reduction of acetaldehyde to ethanol [36, 37]. *ADH* genes are involved in a wide range of metabolic processes including hypoxia (CH_3_CHO detoxification) [2], C-balance (PDH bypass) [38, 39], and scent [40, 41]. This study identified and analyzed the evolution and function of ADHs.

### Evolution of *ADH* gene family in plants

We identified 151 *ADH* genes, including 43 in five dicots, 95 in nine monocots, 7 in *A. trichopoda*, and 6 in two bryophytes. No *ADH-P* gene was identified in algae (*C.s crispus*, *C. merolae*, and *G. sulphuraria*). However, one *Class III ADH* gene was obtained from all these three species. The *ADH-P* gene family originated from *Class III ADH* [2]. It is thought that the *ADH-P* gene may only be present in terrestrial plants. The copy number of the *ADH* gene in plants varies widely but mostly ranges from 6 to 10. The largest number (32) was found in *S. spontaneum*. *P. patens* had the smallest number (2). The results indicated that the *ADH* gene family has undergone several rounds of gene duplication events during its evolution. The phylogenetic tree suggests that the origin of the *ADH* gene can be traced back to a common ancestor of lower plants and higher plants before their divergence. The formation of actual multigene families in plants was probably driven by duplication events.

As the phylogenetic tree of the examined plants indicated, the *ADH* gene families were phylogenetically clustered into four groups (Groups A-1, A-2, B-1, and B-2). The duplication event (D1) resulted in the separation of Rhodophyta and other embryophytes (Figure S1). The Group A-1 and A-2 only contained all the representative plants whereas the Group B-1 and B-2 only contained the seed plants. The results implied that B-1 and B-2 may have divided before the divergence of seed plants, and the mosses might have lost *ADHs* belonging to these groups during their evolution. WGDs are considered to be important evolutionary events [42]. Some WGDs, associated with the origin of the recent common ancestor of extant angiosperms (ε), pan-core eudicots (γ), and monocots (ρ), have been revealed by integrated synteny, age estimates of gene duplication, and phylogenomic analysis [43, 44]. WGD, occurring at 150–270 Mya, is considered to be the most recent evolutionary event and produced terrestrial plants, which are the common ancestors of current angiosperms [43–45]. *A. trichopoda* is strongly supported as the single living representative species of the sister lineage to all other extant flowering plants. It provides a unique reference for inferring the genome content and structure of the most recent common ancestor of living angiosperms [46]. In B-1 and B-2, the divergence appears to have occurred before the emergence of *A. trichopoda*, indicating that all these *ADH* genes experienced duplication. There is a clear separation between the monocot and eudicot lineages in the four groups. These results suggest that additional duplications occurred subsequently to the angiosperm radiation. It also suggests that before monocots and eudicots split (165 Mya), the *ADH* genes may have undergone divergence or functional specialization.

### Comparative genomics of *ADH* gene family in *S. bicolor*, *S. spontaneum* and R570

Within the three focal genomic references (*S. bicolor*, *S. spontaneum* and R570), the genomic features of the predicted ADHs specifically with regard to their motif, gene structure, chromosomal arrangement, duplicated types, and synteny were investigated.

Substantial differentiations may have occurred during the evolution of gene families. The divergence of their protein sequences is usually used to study their phylogenetic topology and reveal their evolutionary process. Similarly, gene structure is commonly used to investigate and infer evolutionary processes [47, 48]. In this study, the gene structures were analyzed to study the structural characteristics and evolution of the *ADH* genes (Fig. 3). Among *S. bicolor*, *S. spontaneum* and R570, the intron number of *ADH* genes ranged from 0 to 16, and 26 of the 47 *ADH* genes had nine introns. In the common ancestor, the standard number of introns in plant *ADH* genes is nine, and these are located at similar positions in *ADH* genes within the entire plant kingdom [2]. In melon, Jin et al. (2016) found that the intron numbers of *ADH* genes ranged from 2 to17 per gene [5]. In *Gossypium*, the ADHB, C and D loci carried genes with nine introns [49]. We speculate that a basic gene model consisting of nine introns was the structure of ancestral *ADH* genes. Based on phylogenetic analysis, we infer that the age, in duplicated descending order, for the four groups is A-1, A-2, B-1, and B-2 (Figure S1). Compared to other groups, the exon/intron organization in B-2 genes is more diverse, which may because ADHs in Group B-2 mainly originated from recent single-gene duplications rather than ancient WGD events. The number of introns of *ADH* genes in B-2 varied, suggesting that the insertion and/or loss of introns, the gain of an intron or fusion of exons all might have occurred during evolution.

Chromosome distribution analysis demonstrated that *ADHs* (*SbADHs*, *SsADHs*, and *ShADHs*) in the three reference genomes were unevenly distributed on chromosomes 1, 2, 4, and 5, respectively. We suggest that the chromosome distributions in these three plant species are conserved. Synteny analysis showed that there are at least four collinear blocks between each pair in the three species. Previous studies showed that inversions and rearrangements could be predicted among chromosomes Ss2 (A, B, C, and D) of AP85–441 and Sb5 of sorghum [27]. However, SsADH3a, SsADH3b, SsADH4a, SsADH4f, and SbADH2 were not located in the rearranged chromosomal regions. Rody et al. [22] found that the genes that are located in the non-arranged chromosomal regions between AP85–441 and sorghum may represent conserved sources of disease resistance in sugarcane and sorghum. The disease resistance function of ADH requires further study.

Dispersed, tandem, proximal, and WGD/segmental duplications were identified among the *ADH* genes within the three focal genomic references. These four gene duplication events guided the evolution of gene families encoding proteins at the gene and chromosomal levels [50]. A tandem duplication is the result of a single unequal crossing event and/or multiple iterations during DNA repair [51]. Many plant gene families involved in plant disease resistance and glucosinolate biosynthesis have copy number variations caused by tandem duplication [52]. In our study, tandem duplication appeared to be the major force in the gene expansion of *SbADHs*. Dispersed duplication as a small-scale gene duplication event, also played an important role in gene family expansion [53]. About 67% of the *ShADHs* in R570 were expanded by dispersed duplication. WGD is a large-scale duplication event. Almost all angiosperms have experienced at least one WGD [43]. In *S. spontaneum*, more than half of *SsADHs* were related to WGD/segmental duplication. The results showed that tandem duplication, dispersed duplication, and WGD/segmental duplication were the main driving forces for the expansion of the ADH gene family in *S. bicolor*, *S. spontaneum* and R570, respectively.

### The function of *ScADH3* under various stresses

*ScADH3*, with an ORF length of 1137 bp, was cloned by RT-PCR amplification. This gene encodes a 379 amino acid polypeptide. Sequence analysis showed that ScADH3 contained a Zn1-binding signature, the Zn2 structural motif and the NADPH-binding domain motif (so-called Rossmannfold). The results suggest that ScADH3 appears to be a zinc-dependent ADH belonging to the plant ADH protein family and the medium-chain dehydrogenase/reductase (MDR) superfamily [54, 55].

qRT-PCR analysis suggested that *ScADH3* responds to a variety of stresses. Pathuri et al. [56] found that *Blumeria graminis f.sp. hordei* can induce an increase of ADH enzyme activity. Under *S. scitamineum* attack, transcripts of *ScADH3* in this study were induced at 24 h and 48 h. Infection by pathogenic bacteria can possibly induce the expression of *ADH* genes and increase ADH enzyme activity. ABA helps to regulate adaptive responses to stresses [57]. *ADH1* was used as a stress responsive marker gene, especially in the ABA-responsive signaling pathway in *Arabidopsis* [58]. We also found that *ScADH3* was induced by ABA, and its expression pattern was similar to that of the *ADH* gene in *Arabidopsis* [14]. *ScADH3* is also up-regulated under abiotic stresses (PEG, NaCl, and 4 °C). These results are consistent with studies on other species showing that the expression of *AtADH1* is up-regulated by salt, dehydration [18, 36, 59], and cold stress [8, 9, 60]. Based on these results, we believe that *ScADH3* may be involved in the abiotic and biotic stress responses of sugarcane, especially under cold stress, since the expression of this gene was continually upregulated by 4 °C stress.

The *ADH* gene is one of the most common cold-induced genes in cereals and *Arabidopsis* [9]. Considering the qRT-PCR expression analysis results, we performed cold stress experiments in transformed *E. coli* and *N. benthamiana* overexpressing the *ScADH3* gene. Prokaryotic expression analysis showed that *ScADH3* enhanced the tolerance of *E. coli* cells to cold (4 °C) stress. This suggested that even when *ScADH3* is expressed in prokaryotes, it can play a positive role in the response to low temperature stress. In a eukaryotic expression system, after exposure to freezing, transgenic tobacco plants had less foliar damage than wild-type tobacco. The level of ROS is an indicator of the magnitude of stress severity and stress tolerance. A lower level of ROS following stress exposure is an indicator of improved tolerance [61]. Therefore, detecting the level of ROS in plants can increase our understanding of plant tolerance. Histochemical staining clearly showed that, after cold treatment, the transgenic *ScADH3* overexpressing tobacco accumulated less H_2_O_2_ than the WT. Lower ROS levels may explain why transgenic plants have increased ability to withstand damage from cold stress. The balance between ROS generation and scavenging is the major determinant of ROS homeostasis during stress [62, 63]. Hence the transcription levels of three hydrogen peroxide genes (*NtPOD*, *NtSOD*, and *NtCAT*) were detected to study the molecular mechanisms of transgenic and wild-type plants under cold stress. Under cold stress, the transcript expression of two hydrogen peroxide genes (*NtPOD* and *NtCAT*) in the transgenic lines was significantly higher than that in the WT. This suggests that the transgenic plants may be better able to eliminate ROS. This also explains why the accumulation of ROS in transgenic lines is low. In summary, these results indicate that *ScADH3* may control ROS accumulation by regulating antioxidant-scavenging to achieve enhanced cold resistance.

## Conclusions

We identified 151 ADH proteins in 17 plant genomes (five eudicots, nine monocots, one basal angiosperm, and two mosses). Phylogenetic analysis showed that *ADH* genes subfamilies of *ADH* genes underwent distinct gene duplication patterns during angiosperm evolution. Comparative genomics analysis demonstrated the close evolutionary relationship of *ADH* gene families in *S. bicolor*, R570 and *S. spontaneum*. Function analysis implied that *ScADH3* is involved in cold tolerance, which involved modulation of the homeostasis of reactive oxygen species (ROS) by regulating ROS-related genes. These findings reveal evolutionary and functional aspects of the *ADH* genes in sugarcane and identify genes that may be useful for genetic manipulation.

## Methods

### Plant materials and treatments

The sugarcane cultivar ROC22 (*Saccharum* spp. hybrid) was used for gene cloning and gene expression analysis. This was provided by the Key Laboratory of Sugarcane Biology and Genetic Breeding, Ministry of Agriculture (Fuzhou, China).

To study the expression of the sugarcane *ScADH3* gene in response to ABA, abiotic, and biotic stress, uniform four-month-old tissue cultured plantlets of sugarcane were grown in water for 1 week at 28 °C with a 16:8 h (L:D) photoperiod. For biotic stress, ROC22 was inoculated with 0.5 μL of a 0.01% (v/v) Tween-20 suspension containing 5 × 10^6^ smut spores·mL^− 1^; the control was inoculated with 0.01% (v/v) Tween-20 in sterile distilled water [64–66]. Five buds were randomly selected at 0 h, 24 h, 48 h, and 72 h after inoculation. For abiotic stresses, under 25% PEG 8000 and 250 mM NaCl treatments, the samples were collected at 0 h, 6 h, 12 h, and 24 h. For the plantlets under 4 °C treatment, sampling times of 0 h, 6 h, 24 h, and 48 h were established. For exogenous hormone stress, the whole plantlets treated by 100 μM ABA were harvested at 0 h, 3 h, 6 h, and 12 h. Three biological replicates were prepared for each treatment. All of the harvested plant tissues were frozen in liquid nitrogen and stored at − 80 °C until total RNA extraction.

### Identification of *ADH* family genes and conserved residue analysis in plant genomes

Twenty sequenced plant genomes were collected and screened for *ADH* genes to obtain a representation of the major plant lineage (Supplementary Table S7). Among them, 15 plant genomes were obtained from Phytozome (https://phytozome.jgi.doe.gov/) [67]. The genome data of *S. spontaneum* were downloaded from the following link: http://www.life.illinois.edu/ming/downloads/Spontaneum_genome/ [27]. The monoploid reference R570 genome came from the Sugarcane Genome Hub (http://sugarcane-genome.cirad.fr/) [26]. Three genomes of Rhodophyta were collected from Ensembl (http://plants.ensembl.org/index.html) [68]. The HMM profile of ADH_N (PF08240) was downloaded from the Pfam protein family database (Pfam; http://pfam.sanger.ac.uk/) [69]. Using HMMER v3 [70] with the Hidden Markov Model (HMM) corresponding to the raw ADH_N, ADH protein candidates from the 20 plant genomes were obtained. The first transcript isoform was selected if two or more transcripts were annotated for the same gene from alternative splicing. Putative ADH protein sequences were submitted to CDD (https://www.ncbi.nlm.nih.gov/cdd) [71] to confirm the domain. Finally, sequences with complete domains were preserved. ExPASy (http://web.expasy.org/protparam/) was used to predict the basic properties (MW, pI, GRAVY, and instability index) of these *ADH* genes encoded proteins. Subcellular localizations were predicted by WoLF PSORT (https://wolfpsort.hgc.jp/).

### Multiple sequence alignment and phylogenetic analysis

MUSCLE v3.7 [72] was used to conduct a protein multiple sequence alignment (MSA) analysis with default parameters. The MSA was used to generate a ML phylogenetic tree with the RaxML-HPC tool [73]. The bootstrap value was set to 1000 repetitions. The resulting treefile was visualized with FigTree version 1.4.4 (http://tree.bio.ed.ac.uk/software/figtree/) and EvolView (https://www.evolgenius.info/evolview/#login) [74].

### Chromosomal distribution and gene duplication

MapGene2Chrom (MG2C) software (http://mg2c.iask.in/mg2c_v2.1/) was applied to map the chromosomal positions of the *ADH* genes in *S. bicolor*, R570 and *S. spontaneum*. Synteny block and gene duplication events were determined and analyzed by the MCScanX with the default parameters [75]. The Ka/Ks was calculated by TBtools [76].

### Determination of protein motif distribution and gene structure

The online MEME program (http://meme-suite.org/tools/meme) (Bailey et al., 2006) was used to analyze protein motifs with the parameters of maximum motif number with 20, minimum motif width with 6, maximum motif width with 50, and distribution of motif occurrences with zero or one per sequence. The protein motifs and gene structures were drawn by iTOL (https://itol.embl.de/).

### Gene isolation and protein structure analysis

The total RNA of all samples was extracted using TRIzol® Reagent (Invitrogen, Carlsbad, CA, USA). According to manufacturer specifications, first-strand cDNA synthesis was synthesized using the RevertAid First Strand cDNA Synthesis Kit (Fermentas, Shanghai, China). For qRT-PCR analysis, the Prime-Script™ RT Reagent Kit (Perfect For Real Time) (TaKaRa, Dalian China) was used to perform first-strand cDNA synthesis.

The sequence of a putative *ScADH3* was obtained from our previous RNA sequencing data in sugarcane [77]. The primers for *ScADH3* were designed by the NCBI primer designing tool (http://www.ncbi.nlm.nih.gov/tools/primer-blast/) (Supplementary Table S8). Based on the specifications for *Ex* Taq (TaKaRa, Dalian, China), RT-PCR reaction was constructed. The first-strand cDNA of ROC22 was chosen as the amplification template. The amplification reaction was 94 °C for 4 min; 94 °C for 30 s, 58 °C for 30 s, 72 °C for 1 min 20 s, 35 cycles; and 72 °C for 10 min. Gel-purification of the production of RT-PCR and cloning into pMD19-T vector (TaKaRa, Dalian, China) was then conducted. The recombinant plasmid pMD19-T-*ScADH3* was transformed into *E. coli DH5α* competent cells and sequenced (Sangon, Shanghai, China). Clustal Omega (https://www.ebi.ac.uk/Tools/msa/clustalo/) was used to calculate the percent identity matrix between 6 ShADHs, 32 SsADHs and ScADH3. The primary and secondary structure analysis of ScADH3 was predicted by ExPASy (http://web.expasy.org/protparam/) and prabi (http://npsa-pbil.ibcp.fr/cgi-bin/npsa_automat.pl?page=npsa_gor4.html), respectively.

### Expression pattern under *S. scitamineum*, ABA, NaCl, PEG, and 4 °C stresses

The relative expression levels of *ScADH3* under different exogenous stresses were detected using qRT-PCR. Beacon Designer 8.12 software was employed to design the qRT-PCR primers of *ScADH3*. The glyceraldehyde-3-phosphate dehydrogenase (*GAPDH*) gene was selected as the reference gene [78, 79]. The qRT-PCR reaction system (SYBR Green Master Mix: 10 μL, 10 μM forward and reverse primers: 0.8 μL, 20 × diluted cDNA template: 1.0 μL, and sterile distilled water: 7.4 μL) was constructed with reference to the manual of SYBR Green Master Mix (TaKaRa). The reaction procedure was as follows: 50 °C for 2 min, 95 °C for 10 min, 40 cycles of 95 °C for 15 s and 60 °C for 1 min. The 2^-ΔΔCt^ method was used to normalize the relative expression level of qRT-PCR data [80]. Each of the samples had three biological replicates. Three technical replicates were performed. All primers used in qRT-PCR are listed in Supplementary Table S8. Data Processing System v9.50 software (China) was used to conduct the statistical analysis. Data were expressed as the mean ± standard error (SE), Significance (*p* < 0.05) was calculated using one-way ANOVA, followed by Duncan’s new multiple range test.

### Cold stress tolerance assay using transformed *E. coli* BL21 (DE3) cells

Prokaryotic expression in *E. coli* BL21 (DE3) cells is usually employed to study the function of genes under abiotic stresses [64, 81–84]. In this study, the prokaryotic expressive vector of pEZYHb-*ScADH3* was constructed by LR ClonaseTM II Enzyme Mix (Invitrogen) and transformed to *E. coli* BL21 (DE3) competent cells. *E. coli* BL21 with the empty vector pEZYHb was used as the control. When *E. coli* BL21 cells containing different vectors were grown to OD_600_ of 0.6, 1.0 mmol L^− 1^ isopropyl β-D-thiogalactoside (IPTG) was added to induce protein production. After continuous growth at 37 °C for 12 h, we adjusted the concentration of the culture to OD_600_ of 0.6 and used LB medium to dilute the samples by 10^− 3^- and 10^− 4^-fold. Finally, 10 μL from each of the 10^− 3^- and 10^− 4^-fold dilutions of the sample was spotted on LB agar plates. For the cold tolerance assay, samples were spotted on LB agar plates, and they were placed at 4 °C in darkness. After 3 days, 7 days, and 14 day at 4 °C, the plates were cultured at 37 °C overnight and then photographed.

### Cold tolerance assay of *N. benthamiana* overexpressing *ScADH3* gene

The overexpression vector pEarleyGate 203-*ScADH3*, which contained the 35S promoter, was constructed by gateway cloning technique and then transformed into *Agrobacterium* strain GV1301 competent cells. For transformation into *N. benthamiana*, the leaf-disc method was employed [85]. Transgenic lines were selected by glufosinate ammonium and further confirmed by RT-PCR amplification.

Seeds of three transgenic tobacco lines (OE) at the T2 generation were germinated and selected on 1/2 Murashige and Skoog (MS) medium with glufosinate ammonium and the WT were germinated on 1/2 MS medium. One month later, the seedlings were transferred to soil pots and kept in a growth chamber (16:8 (L:D) photoperiod, and 25 °C). The cold treatment method was similar to that used by Geng and Liu [61] with slight modifications. We used two-month-old tobacco plants (OE and WT) that were subjected to − 2 °C for 3 h. The leaves were collected after cold treatment for phenotypic observation, physiological measurement and gene expression analysis. To characterize the function of *ScADH3*, the phenotypic variations were observed by comparing the wild-type and the transgenic plants. We judged the tolerance of tobacco to cold stress based on the water-soaking degree of leaves [61]. Histochemical staining with 3, 3′-diaminobenzidine (DAB) was used to detect the accumulation of H_2_O_2_ in tobacco leaves [86, 87]. Briefly, freshly prepared solutions of 1 mg ml^− 1^ DAB (in 50 mM potassium phosphate, pH 3.8) were used to incubate the leaves. After incubation for 12 h in darkness at room temperature, the chlorophyll was removed with 75% ethanol in a boiling water bath, and the leaves were then photographed. Transcript analyses of the three tobacco hydrogen peroxide-related genes (*NtPOD*, *NtSOD*, and *NtCAT*) were conducted on the treated *N. benthamiana* leaves according to Geng and Liu [61], and the *NtUbiquitin* was treated as a reference gene [61]. The 2^-ΔΔCt^ method was used to normalize the relative expression level of qRT-PCR data [80]. RT-PCR was used to detect whether *ScADH3* had been overexpressed in *N. benthamiana*, with the RNA of treated leaves and *ScADH3* specific primers. The *NtEF1-α* was treated as control. The RT-PCR procedure was 94 °C for 4 min; 94 °C for 30 s, 58 °C for 30 s, 72 °C for 1 min 30 s, 35 cycles; and 72 °C for 10 min. Each of the samples had three biological replicates. Three technical replicates were performed. All primers used in qRT-PCR are listed in Supplementary Table S8. All experiments had three biological replicates. All samples, after treatment, were frozen in liquid nitrogen and stored at − 80 °C.

## Supplementary information

**Additional file 1: Figure S1.** Phylogenetic relationship of *ADH* gene family in major groups of plants.

**Additional file 2: Table S1.** The detailed information of *ADH* genes included in this study. **Table S2.** The gene type of *ADH* genes in *S. bicolor*, R570, and *S. spontaneum*. **Table S3.** One-to-one orthologous relationships between *S. bicolor*, R570, and *S. spontaneum*. **Table S4.** Percentage of identity between 6 ShADHs, 32 SsADHs, and 1 ScADH was calculated using Clustal Omega. **Table S5.** Primary structure analysis of ScADH3. **Table S6.** Secondary structure analysis of ScADH3. **Table S7.** Sources of *ADH* genes from sequenced plant included in this study**. Table S8.** Primers used in this study.

## Data Availability

The data supporting the conclusions of this article are within the paper.

## Abbreviations

ADH: Alcohol dehydrogenases
WGD: Whole-genome duplication
ROS: Reactive oxygen species
ABA: Abscisic acid
PEG: Polyethylene glycol
NaCl: Sodium chloride
SA: Salicylic acid
MeJA: Methyl jasmonate
MW: Molecular weight
pI: isoelectric points
GRAVY: Grand average of hydropathicity
Mos: Mosses
Bal: Basal angiosperms
Mon: Monocots
Eud: Eudicots
MDR: Medium-chain dehydrogenase/reductase
HMM: Hidden Markov Model
MSA: Multiple sequence alignment
MG2C: MapGene2Chrom
MCScanX: Multiple Collinearity Scan toolkit
IPTG: Isopropyl β-D-thiogalactoside
OE: Transgenic tobacco lines
WT: Wild type
MS: Murashige and Skoog
DAB: 3, 3′-diaminobenzidine
GAPDH: Glyceraldehyde-3-phosphate dehydrogenase
RT-PCR: Reverse transcription-polymerase chain reaction
qRT-PCR: quantitative reverse transcription polymerase chain reaction
SE: Standard error
