# Supplementary material for: The alcohol dehydrogenase gene family in sugarcane and its involvement in cold stress regulation
Source: BMC Genomics. 2020 Jul 29;21:521. doi: 10.1186/s12864-020-06929-9 (PMC7392720; doi:10.1186/s12864-020-06929-9)
Supplement: Supplementary file 1 — Additional file 1: Figure S1. Phylogenetic relationship of ADH gene family in major groups of plants. [file 12864_2020_6929_MOESM1_ESM.pdf]

The alcohol dehydrogenase gene family in sugarcane and its involvement in cold stress regulation

Weihua Su[1,2], Yongjuan Ren[1,2], Dongjiao Wang[1,2], Yachun Su[1,2], Jingfang Feng[1,2], Chang Zhang[1,2], Hanchen Tang[1,2], Liping Xu[1,2], Khushi Muhammad[3], Youxiong Que[1,2]*

[1]Key Laboratory of Sugarcane Biology and Genetic Breeding, Ministry of Agriculture, Fujian Agriculture and Forestry University, Fuzhou 350002, China

[2]Key Laboratory of Genetics, Breeding and Multiple Utilization of Crops, Ministry of Education, Fujian Agriculture and Forestry University, Fuzhou 350002, China

[3]Department of Genetics, Hazara University, Mansehra, Pakistan

***Correspondence should be addressed to queyouxiong@126.com**

The full postal address of the submitting author Youxiong Que is as follows: Key Laboratory of Sugarcane Biology and Genetic Breeding, Ministry of Agriculture, Fujian Agriculture and Forestry University, Fuzhou 350002, China

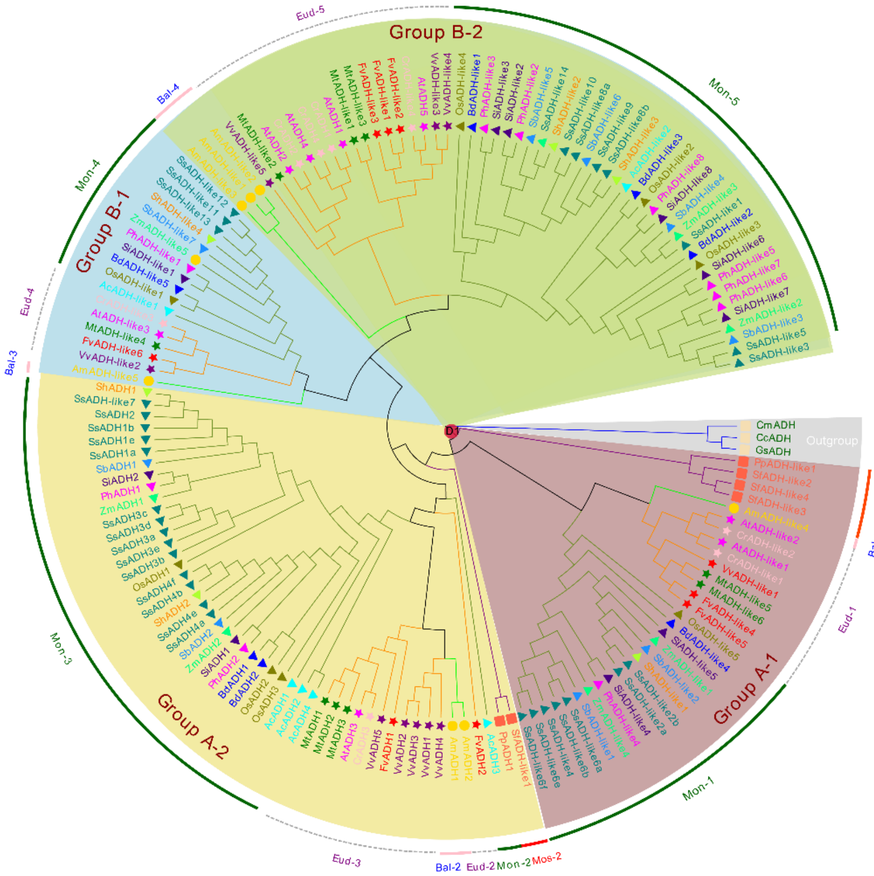

Figure S1. Phylogenetic relationship of *ADH* gene family in major groups of plants. The phylogenetic tree was built using the ML method implemented in RaxML-HPC2 and the Rhodophyta was rooted as the outgroup. There are four major subgroups (A-1, A-2, B-2, and B-2). Gene lineages composed of eudicots (Eud), monocots (Mon), basal angiosperms (Bal), mosses (Mos), and Rhodophyta are labeled on the outside of the circle.
